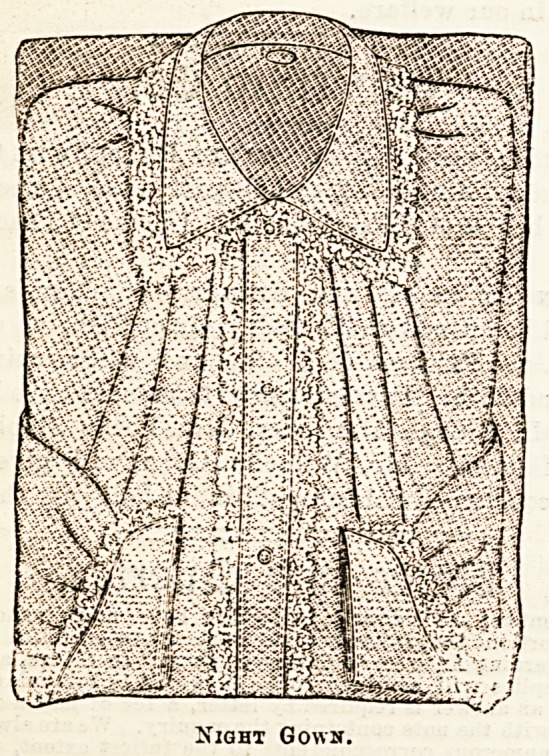# The Hospital Nursing Supplement

**Published:** 1895-06-22

**Authors:** 


### "The Hospital" Nursing Mirror.

Being the Extra Nursing Supplement of "The Hospital" Newspaper.

[Contributions for this Supplement should be addressed to the Editor, The Hospital, 428, Strand, London, W.C., and should have the word "Nursing" plainly written in left-hand top corner of the envelope.]

### News from the Nursing World.

### DISTRICT NURSING AT KENSINGTON.

Her Royal Highness Princess Louise last week attended the annual meeting of the Kensington District Nursing Association, of which she is president. Her Royal Highness was accompanied by the Marquis of Lorne, and the meeting, which was held in Kensington Town Hall, was very well attended, the Rev. and Hon. E. Carr Glyn being in the chair. The staff of the association consists of a superintendent and six trained nurses, whose services are given gratuitously to the sick poor in their own homes. The Marquis of Lorne, in seconding the motion for the adoption of the report, bore eloquent testimony to the good work done by the nurses, urging on all present that it was the duty of the inhabitants of Kensington to take care that the work was not crippled by any want of funds.

### UNIVERSITY COLLEGE HOSPITAL NURSES.

The pleasant excursion annually arranged for the nursing staff of University College Hospital by the secretary, Mr. Nixon, took place last week, the whole expense being defrayed as usual by special subscriptions given by members of the hospital and medical committees, the council of the college, and other supporters of University College Hospital. A number of the sisters and nurses were driven down to Richmond, and thence went in an electric launch on the river. The weather was all that could be desired. Lunch, tea, and supper were provided at the Castle Rooms, and the guests returned home in the evening, having added another pleasant experience to the record of the entertainments so successfully organised for their enjoyment by Mr. Nixon.

### HELP THOSE WHO HELP THEMSELVES.

"Where are you going for your holiday?" is the question heard on all sides just now, and many are the plans made for spending the all-too-short period advantageously. With the words "Rest" and "Change" in our ears, it is but natural that we should think of those amongst our sisters who long for them in vain. To enable such as are incapacitated by the effects of recent illness to get the required change of air and scene is the work which The Hospital Convalescent Fund undertakes. Any nurse is eligible for its aid whose own means do not suffice for the change which the doctor considers essential to restore her health. To mend up broken health and to give needful rest to overworked nurses are the main objects of The Hospital Convalescent Fund, which does not attempt to provide for chronic cases nor those which need active medical treatment. To meet the increasing demands on our modest little fund more subscriptions are needed. We therefore appeal with confidence to those readers who have helped in the past, as well as to the new friends who constantly join our circle. Address Honorary Secretaries The Hospital Convalescent Fund, 428, Strand.

### TRAINING AT TOTTENHAM.

In the training of deaconesses at the Tottenham Hospital for "working, teaching, and nursing sisters," the instruction allotted to the nursing department appears to have been hitherto most inadequate. As a result of the publicity now given to the subject by the press, it may be hoped that the council will realise that probationers, whether lay women or prospective deaconesses, can only be duly instructed by fully-trained nurses.

### AN EXCELLENT EXAMPLE.

A courteous invitation was issued by the St. Pancras Infirmary Visiting Committee for the 21st inst., when they asked those interested in nurses and nursing to make a general inspection of their institution. The Nurses' Home opened last winter, and the well-kept wards must prove equally encouraging to those who have the welfare of the sick poor at heart. We commend the action of the St. Pancras Committee to the notice of all guardians, and we wish all success to the great institution on Highgate Hill where a three years' course of training was introduced many years ago.

### NURSING IN COTTAGE HOSPITALS.

Whether in a remote country district or in a neighbourhood such as Wood Green, where a new cottage hospital was opened last week, the question of the nurses demands equal attention. A kindly measure of local interest may generally be reckoned on both for the pretty new building and also for the individual patients. Perhaps from lack of practical knowledge of the requirements of skilled nursing there is rarely due consideration shown by the community towards the nurses. Their rooms are made pretty, and they themselves are often treated with courtesy by the committee, but the duty of the latter towards them does not end thus. The nurse-matron placed by them in charge of six, eight, or ten patients is responsible to the doctor for the care and feeding of the sick, and to the committee for the cleanliness of the whole house. She must deal with all emergencies, including stoves that "won't draw," as well as with sudden illness or accidents. The cooking of the establishment has to be personally supervised and often partially performed by her. The store of linen must be kept in order and the laundry economically managed. It is, therefore, not necessary for the beds to be all full to make the matron's hands busy. Two or three serious cases, or even one who is delirious, of course necessitate night watching, and this absorbs the services of a probationer, if there is one. Wherefore it cannot be too often urged on all who organise new cottage hospitals, or manage those already in existence, that a bad case makes a night nurse necessary. It may not be desirable to increase the nursing staff permanently, but it should be an invariable rule that an extra nurse is temporarily secured when night duty is necessary. It is mistaken economy to postpone this measure until the nurse-matron breaks down from doing double duty.

### THE TROUBLES AT MACCLESFIELD.

In a preliminary discussion regarding the appointment of a new Matron at Macclesfield Infirmary, the Mayor is reported to have remarked that "he hoped in their next appointment they would meet with some one who would have the good opinion of everyone." The meeting at which this sanguine hope was expressed did not, however, show the existence of much present unanimity at this institution. Amongst other subjects, on which different opinions prevailed, was the condition of the lock on the matron's door! Exception was taken by some members of the committee to Miss Wingfield having left a sealed tape on her door during her absence. It has since been asserted that this was merely done because the lock did not act, but instead of deciding the question once for all by a personal inspection of the door, "the meeting broke up in disorder." Although regrets were expressed by some members of the committee at the alleged discourtesies to Miss Wingfield, no definite explanations was arrived at concerning the accusations of "friction" which had been circulated. A letter from Miss Wingfield in the *Macclesfield Courier* asserts that not only was everything in her bed-room turned out in the presence of the two house surgeons and the secretary, but locks were forced and her private papers and other effects disturbed during her absence. In another column we print a letter from a lady describing the improvements which have been introduced by Miss Wingfield into the nursing arrangements at the Macclesfield Infirmary.

### A FETE AT COLD ASH.

A successful Floral Fête was held the other day at Cold Ash in aid of the building fund of the Children's Cottage Hospital. Lady Jeune opened the fête, which was largely attended during the day. The little hospital is a very pretty one, and contains twenty patients, who do credit to the skill and kindness lavished on them.

### A SATISFACTORY ARRANGEMENT.

At the annual meeting of the South Shields Nursing Guild it was shown that the committee had every reason to congratulate themselves on having secured a fully-trained nurse for the poor. Up to last January the sick had been visited by the associates of the St. John's Ambulance Association, "but," said the Honorary Secretary, "it was unmistakably evident that if the work was to be thoroughly carried out it was essential to have one whose whole time could be given to the arduous work of nursing." Since February 5th the nurse engaged by the committee had paid 909 visits to 42 patients, and the association is not likely to regret the provision thus made for its poor brethren.

### QUEEN'S NURSES AT DARLINGTON.

The weather was favourable for the sacred concert held according to custom in aid of the funds of the Queen's nurses at Darlington on a recent Sunday afternoon. There was a large attendance in the Public Park, the Mayor and Mayoress being amongst the audience. One or two short speeches showed the keen interest taken locally in this branch of the Queen's Jubilee Institute, and £16 10s. resulted from the collection made afterwards.

### THE WORK OF A WOMAN.

The death of Mademoiselle Leontine Nicolle, at seventy-two years of age, was lately announced in Paris. Most valuable work was done by this lady in the school attached to the Salpêtrière Hospital, where she took over in 1850 the superintendence of idiot and epileptic girls. To her devoted care, it is said, many recoveries were considered to be due, and undoubtedly a great improvement in the general condition of these afflicted persons took place under Mademoiselle Nicolle's intelligent sway. In recognition of her services she was presented by the French Academy with the Montyon prize and the "Palmes Académiques," and later on she received the Cross of the Legion of Honour at the hands of President Carnot.

### WHO IS RESPONSIBLE?

Authority at Armagh appears to be somewhat curiously distributed, for whereas the Board takes care to have a candidate passed by their doctor before her admission for three months' probation, they can entirely ignore his advice at the conclusion of that period. The doctor (according to a report in the local press) wrote to the Guardians declining to sanction a probationer's engagement for a further term of three years, and although his reason for this is not given, it is only fair to believe that he considered he had a sufficient one. It was, however, unanimously decided by the Board to sanction the permanent appointment of the probationer, the House Committee, it is said, having already expressed approval of this step, although in taking it they put a public slight on the opinion of their own medical officer.

### PENSIONS FOR AMERICAN NURSES.

The steps which have been taken in Philadelphia for the formation of an American Pension Fund for Nurses appear to be viewed with displeasure by a large body of our American sisters. Whether this opposition is merely due to the prejudices with which new departures are generally received, or whether it is more deeply rooted, time will show. The organising committee propose that the movement inaugurated at Philadelphia shall be a national and unsectarian one, and that a charter be obtained for the American Pension Fund. Masseurs, masseuses, and nurses will be eligible for membership up to the age of fifty years. The plan for a bazaar to be held at Philadelphia in the autumn in aid of the funds of the new association appears distasteful to American nurses in other cities, as they think such a project savours too much of charity to coincide with their national independence.

### SHORT ITEMS.

The London Hospital athletic sports took place on the 12th inst. at the club's ground, Lower Edmonton. —The annual meeting of the Affiliated Benefit Nursing Associations was held on June 6th at the residence of Lord Egerton of Tatton, when Miss Broadwood read the report.—The subscribers to the Buckingham Nursing Home held their annual meeting recently.—A street collection, made by the ladies of Camborne, resulted in a sum of £22 being added to the funds of the District Nurse Association.—The Barry District Nursing Association and Accident Hospital received the gate-money taken at the Cadoxton Whit-Monday Fête, amounting to £38.—The report of S. Mary's Cottage (Home of Rest), Whitstable-on-Sea, shows that five church workers availed themselves of it last year.

### Elementary Anatomy and Surgery for Nurses.

By W. McAdam Eccles, M.B., M.S., F.R.C.S., Lecturer to Nurses, West London Hospital, &c.

### XXII.—SURGICAL AFFECTIONS OF THE RESPIRATORY PASSAGES.

Foreign bodies may occur in the nose, larynx, trachea, or bronchi. Beads, pebbles, and the like are not infrequently inserted by children into the nostril. The child may be so frightened at the circumstance as to make no reference to it, but sooner or later a fœtid discharge will come from the nose, and this should always lead to the suspicion of the presence of a foreign body. In the treatment of such a case a careful examination should be made, and, a body having been revealed, the patient should be told to close the opposite nostril and forcibly expire through the affected one. This failing to dislodge the offending substance, an attempt to remove it by forceps is advisable.

A foreign body impacted in the larynx is a much more formidable condition of affairs, for it leads to much spasm of the glottis, and resulting dyspnœa, or difficulty in breathing. There will be, in addition, a change of tone in the voice, cough, and, after a time, severe inflammation of the mucous membrane. An attempt may be made to hook the body out by the finger if it be felt lodged above the vocal cords; if this be impossible, a surgeon will have to make an opening into the larynx or trachea in order to relieve the dyspnœa.

The trachea or a bronchus may be the seat where a foreign body may be fixed, or found still movable. Such cases require immediate treatment by a surgeon.

Inflammation of the larynx or laryngitis is a somewhat common affection, resulting in most instances, when of the acute form, from cold, violent exertion of the voice, or spread of inflammation from surrounding parts. Chronic laryngitis on the other hand may also be due to similar causes, but in addition it is caused by certain diseases, as, for instance, infection by the tubercle bacillus. The symptoms of laryngitis are usually clear. There is hoarseness, cough, pain, and often some amount of dyspnœa. The treatment varies, according as the case is one of acute or chronic laryngitis. Rest, particularly abstinence from speaking, giving moist air for inspiration by means of steam from a kettle, and inhaling certain vapours.

There are two other forms of laryngitis which must be noticed, viz., the œdematous and the diphtheritic. Œdematous laryngitis or œdema of the glottis is present when the mucous membrane of the upper part of the larynx becomes much swollen by the inflammatory effusion which leads to very urgent dyspnœa. It is commonly the outcome of scalds of the larynx, produced by a child drinking very hot water, or of the stings of insects. Tracheotomy, or the making of an opening into the trachea, has usually to be performed in these cases. Diphtheritic, or membranous laryngitis, is unfortunately by no means a rare affection, and occurs most frequently in young children, who from the smallness of the parts can ill brook any obstruction to their respiratory passage. In addition to this condition it must be remembered that diphtheria is essentially a disorder of the whole body, and that a patient may die from the poison of the disease, altogether apart from any interference with respiration. Diphtheria is characterised by the formation of a whitish yellow false membrane upon the surface of the soft palate, tonsils, and often extending to the larynx and trachea. Owing to the narrowing thus produced an alteration in the cry, voice, or cough is soon noticeable, and this is rapidly followed by dyspnœa. During the violent efforts of the little patient to inspire air, it will be seen that the soft parts of the chest wall, as for instance between the ribs, recede, producing what is called recession. This is an important sign for a nurse to notice and report. When dyspnœa becomes urgent relief may be given in a number of cases only by the operation of tracheotomy. This will often have to be performed very promptly, but haste and flurry should ever be avoided. It is the nurse's duty to see that she holds herself in readiness to assist and act with the surgeon in a manner exhibiting complete presence of mind. After the operation has been completed efficient and careful nursing will be needed to bring about a satisfactory ending to the case. As soon as the patient has been returned to his cot he should be kept warm, and often a steam kettle is allowed to play into the tent arranged around the cot. Personally I think there are many objections to this particular line of treatment. Very persistent watching of the little sufferer is requisite, and a nurse should thoroughly understand what symptoms are dangerous ones, and what points she is particularly to attend to. The management of the tube is all important. A silver tube will probably have been introduced into the trachea, consisting of an outer and an inner portion, the latter projecting a little beyond the lower end of the former. The outer tube has to be kept securely fastened by a tape tied round the neck. Dealing with the inner tube will test the nurse's capability to the utmost. This will have to be frequently but carefully removed, in order to cleanse its interior from the mucus which collects in it. This is best done by placing the tube in warm carbolic lotion and using a brush which can be passed through the tube. Another, and often difficult, task to be accomplished is feeding the patient. At first the liquid food ordered will have to be given by means of a tube passed through the nose and the pharynx and on into the stomach. The utmost patience will be needed in performing this feeding. Later, when food is allowed by the mouth, the nurse must observe whether the child coughs while swallowing, or if there is any other indication that the nutrient is wrongly entering the air passages.

Lastly, in dealing with cases of diphtheria it is highly necessary that the attendant should take the utmost care in self-preservation by avoiding unnecessary risks of infection. Two good rules to follow are, never hold your head over a child when it is coughing, and never under any circumstances kiss your patient.

Empyema is the term used for the presence of pus in the pleural cavity. The pus is usually evacuated by an opening made in one of the intercostal spaces, and the treatment of the case afterwards requires great care and patience. A great deal of discharge flows through the drainage tube, and it will be the nurse's duty to put extra dressing over the part if that applied by the surgeon have become saturated.

### Appointments.

Hinckley Cottage Hospital.—Miss Harrison has been appointed Matron of this hospital. She was trained at the Guest Hospital, Dudley, was nurse at Oldham Infirmary, and afterwards district nurse at Petworth for three years. She appears well qualified for a post in which we wish her every success.

Macclesfield General Infirmary.—Miss Clara E. Bowman has been appointed Matron of this infirmary. She was trained at York County Hospital, was Head Nurse at Yarmouth Hospital for two years, Night Superintendent at Glasgow Royal Infirmary for two years, and Matron of Great Yarmouth New General Hospital for four years. We wish her success in her new work.

### Minor Appointments.

St. Mary Abbots Infirmary, Kensington. — Miss Margaret Ritchie has been made Sister of the Maternity Wards at this infirmary. She was trained at the Radcliffe Infirmary, Oxford, and afterwards did private nursing for five years in the same city. After obtaining the L.O.S. diploma Miss Ritchie worked as a midwife at Sandown, Isle of Wight, for over a year, and takes from thence to her new appointment the good wishes of many friends.

### Hospital Sunday.

South Place Chapel and Institute, Finsbury.—The service arranged by the South Place Ethical Society for Sunday, 16th inst., was conducted by Miss Honnor Morten. A reading from George Eliot's "Scenes of Clerical Life" and various anthems and hymns preceded the discourse on "Life in a London Hospital." Miss Morten spoke of the intense interest surrounding hospital life, and making it stand absolutely alone. The incessant battle silently carried on between life and death, the fight against disease, the great issues ever at stake—all these made it impossible for those who had once shared in the work of a hospital ever to forget. The outward effects in a ward were those of light and of brightness, the spotless whiteness and peace were very beautiful. Miss Morten suggested that the funds of institutions would be materially increased by opening the hospital chapels to the public, and exhibiting the patients and workers of whom the congregations are composed. The sick children who "live in the present" and enjoy it, the precocious lads and pathetic old women were in turn described by Miss Morten. The latter, loth to go back to their own crowded rooms, seldom appreciated the doctor's permission for them to sit up for an hour. To his remark, "You don't want to become bedridden, grannie?" the response might be, "Oh! no, sir," although, to tell the truth, there is nothing the poor old woman would like better than to become bedridden in her present comfortable quarters. Passing on to the daily round of work in hospital, Miss Morten gave a graphic description of the routine of the day and the night, and pleading the cause of the hospitals, and appealing for support, she impressed upon her hearers the need "to recognise that we are our brother's keeper." Whatever might be said of the survival of the fittest, of the mystery of pain, of a time when there will be no hospitals, "while they exist we must support them in the living present." The alleviation of pain being costly, Miss Morten pleaded not only for money to provide it, but also for personal interest in institutions, urging those present to visit hospitals, and especially the small ones, which wanted watching.

### Royal British Nurses' Association.

We are informed by the Secretary of the Royal British Nurses' Association that active measures are being taken to organize a lending and reference library for the use of members at the offices, 17, Old Cavendish Street, a project long since suggested, but only now within measurable distance of being realized. It is confidently anticipated that the library will be in working order early in the autumn, prior to which all members will be officially informed of the scope of its contents, the regulations to be observed, and other details connected with its management.

The Annual Meeting.

The annual meeting will this year offer special attractions to members, in consequence of the gracious intention of H.R.H. the President to hold a reception in the afternoon at the Queen's Hall, Langham Place, to include a concert, in which the Princess will take part.

### National Society for the Prevention of Cruelty to Children.

Under the patronage of her Majesty the Queen, this society has long been well known, both by its list of Royal and noble supporters and for its record of work. The latter is naturally of a difficult and onerous nature, needing much patience and discretion, and the society is to be congratulated on the encouraging report which it has just issued. Information regarding the working of the English, Welsh, Scotch, and Irish branches can be obtained from the secretary at the central office, 7, Harpur Street, London, W.C.

### Medico-Psychological Association.

At the examination held in May, 1895, for the certificate of proficiency in mental nursing, granted by the Medico-Psychological Association of Great Britain and Ireland, candidates were successful from the following asylums: Warwick County Asylum, Hatton; Derbyshire County Asylum, Mickleover; Oxford County Asylum, Littlemoor; Lancashire County Asylum, Rainhill; Stafford County Asylum, Stafford; Stafford County Asylum, Burntwood; Surrey County Asylum, Brookwood; Durham County Asylum, Winterton; Northumberland County Asylum, Morpeth; West Riding County Asylum, Measton; West Riding County Asylum, Wakefield; West Riding County Asylum, Wadsley; London County Asylum, Cane Hill; Joint Counties Asylum, Carmarthen; Borough Asylum, Derby; City Asylum, Bristol; City Asylum, Exeter; Borough Asylum, Plymouth; City of London Asylum, Stone; City of Birmingham Asylum, Rubery Hill; Northumberland House Asylum, London; The Retreat, York; Coton Hill Asylum, Stafford; Warneford Asylum, Oxford; Hoxton House Asylum, London; Bethlem Hospital, London; Holloway Sanatorium, Virginia Water; Broadmoor Asylum, Wokingham; Bethnall House Asylum, London; District Asylum, Larbert, Stirling; Parochial Asylum, Woodilee, Lenzie; Crichton Royal Institute, Dumfries; Royal Asylum, Gartnavel, Glasgow; Royal Asylum, Morningside, Edinburgh; District Asylum, Londonderry; District Asylum, Limerick; District Asylum, Grahamstown, Cape Colony.

Fifty-two candidates (20 males and 27 females) failed to satisfy the examiners.

The following questions were on the paper:—

1. Enumerate the bones forming the skull.
2. State the course the blood takes from the right auricle to the lungs; thence to the heart; from the heart to the general system and back to the heart.
3. Define "Illusion," "Hallucination," and "Delusion."
4. What is meant by "Reflex Action"? Give examples.
5. If a patient cuts a hand deeply how can you tell whether an artery is, or is not, cut? If you feel sure that an artery is cut what would be the first step you would take to arrest bleeding?
6. In taking the temperature of a patient what fallacies may arise? State what precautions you would take to ensure accurate observations.
7. What is meant by (1) Congenital Imbecility and (2) Dementia?
8. Describe the general mental condition in a patient suffering from Dementia?
9. How would you deal with a patient during and after an epileptic fit?
10. Mention some of the mental symptoms which call for early report to the Medical Officer?
11. If you are in charge of a considerable number of patients in a ward, what daily rules would you follow for ascertaining if any of them had escaped from observation?
12. If you are in charge of a patient in a private house, on what points would a daily report be expected from you by the doctor?

The next examination will be held on Monday, November 4th, 1895, and candidates are earnestly requested to send in their schedules duly filled up to the Registrar of the Association (Dr. Spence, Burntwood Asylum, Lichfield), not later than Monday, October 7th, 1895, as this is the last day upon which—under the rules—applications can be received.

### Death in Our Ranks.

We regret to announce the death of Nurse Jessie Jobin, district nurse for Moss Side, Manchester, who died of pleurisy and pneumonia on June 9th. She was trained at the Royal Infirmary, and was a Manchester member of the Guild of St. Barnabas.

### Everybody's Opinion.

[Correspondence on all subjects is invited, but we cannot in any way be responsible for the opinions expressed by our correspondents. No communications can be entertained if the name and address of the correspondent is not given, or unless one side of the paper only be written on.]

### NURSING AT MACCLESFIELD.

A Lady writes: I can speak from personal experience of the work done by Miss Wingfield at Macclesfield. When she went there all was chaos; there was neither nursing for the patients, nor training for the probationers; system and discipline were unknown quantities. The staff nurses were not put at the head of wards, but two acted as day staff, and two as night, taking day and night alternately in the two adult wards, while the large children's ward was entirely nursed by probationers, a three months' one being considered quite capable of taking charge and also of "training" an entirely new probationer. The probationers received a certificate at the end of one year's training; at the end of that time they were often unable to read a medicine-board or understand the most ordinary directions if these were not written in the plainest English. They could not wash a patient in bed, and the heart cases and fractures had their beds made only once in a week. Regular feeding and the administration of stimulants at stated intervals were completely overlooked. The first step Miss Wingfield took was to get experienced heads for the wards and a superintendent of the night-nurses who carried on their training at night. It took three months to compass this, but the reform answered, and almost without exception the probationers proved willing to learn and grateful for being taught. With a trained sister at the head of each ward, and one for the children, the patients gained in comfort, and the nurses in teaching. I have often heard them compare the work since Miss Wingfield came to that in former times. The heavy morning work was strictly supervised, washing and bed-making being taught properly, while in the quiet hours the nurses were every now and then given a set of questions to answer, either on cases under their charge, on anatomy or physiology, or on practical ward work. Miss Wingfield had her reward in six months, in the changed aspect of the place. She increased the course of training to two years instead of one.

### PRIVATE NURSING.

"E. B." writes: There has already been a good deal written on the subject of "Private Nursing," yet I cannot refrain from adding a few remarks in reply to M. G. and "Democrat." Having had twenty years' experience as a private nurse, I have been with families of all grades, from the duke's mansion with a large staff of servants, to small houses with no servant at all. In one of the latter I found only the patient's wife, aged seventy-five, and a boy of thirteen, and there was no water in the house. I do not mean to say it was a desirable position for a nurse to find herself in, but the poor man was ill, requiring skilled attention. I quite agree with "Democrat" as regards the fees; there are many tradesmen who pride themselves on paying high fees. Hitherto I have not found it requires much courage to decline taking my meals in the steward's or housekeeper's room. It is the welfare of her patients which a nurse has most at heart, and on entering any house she certainly prefers to perform her duties in the best possible way. Therefore, she should never fear to speak out when the best interests of the patient might be seriously affected by her not doing so. Suggestions that meals should be served in my own room (which usually joins the patient's) have been received with the greatest courtesy. I find it gives immense influence to be always within call, and thus to know exactly what is happening in the invalid's room. I make a point of getting out every day if possible. Of course, when sitting up every night you must take rest in the day, but in most cases there would be a second nurse. My patients now are chiefly operation cases, and I take sole charge, frequently sleeping in the patient's room. I wish to thank the Editor for his very kind and valuable remarks on "The Meals Question," and for all other proofs of interest in our welfare.

### Where to Go.

Lecture Hall, 10, Upper Avenue Road, Swiss Cottage.—On Saturday, June 29th, at half-past three, a meeting will be held on behalf of "Friedenheim" (Home of Peace for the Dying).

Portman Rooms, Baker Street.—St. Mary's Hospital bazaar will be opened on Thursday, June 27th, at one p.m., by H.R.H. the Princess of Wales, and will remain open on the 28th and 29th. Dramatic entertainments have been promised by Mr. George Alexander, Mr. Yorke Stephens, and Mrs. B. Hannen. Mr. Wilhelm Ganz will give a grand afternoon concert on Saturday, 29th, at half-past three.

### Notes and Queries.

The contents of the Editor's Letter-box have now reached such unwieldy proportions that it has become necessary to establish a hard and fast rule regarding Answers to Correspondents. In future, all questions requiring replies will continue to be answered in this column without any fee. If an answer is required by letter, a fee of half-a-crown must be enclosed with the note containing the enquiry. We are always pleased to help our numerous correspondents to the fullest extent, and we can trust them to sympathise in the overwhelming amount of writing which makes the new rules a necessity. Every communication must be accompanied by the writer's name and address, otherwise it will receive no attention.

### Queries.

(165) *Paris.*—What salary would an English nurse get in Paris, and how obtain work?—*E. J. W.*

(166) *Military.*—How can a trained nurse become an army or navy nursing sister?—*Trained Nurse.*

(167) *Abroad.*—Will you kindly give me information about nursing at Hong Kong and Kimberley, and tell me what dress is suitable there?—*Manchester.*

(168) *Patent.*—Where can I get an improved invalid bed patented?—*P. E.*

(169) *Paralysis.*—Is there any home or institution in or near London where an elderly paralysed lady could be received for moderate payment?—*Jackson.*

(170) *Registration.*—Do two years' hospital training with one year's private work qualify for registration?—*Taunton.*

### Answers.

(165) *Paris* (*E. J. W.*).—We believe the Hollond Institute at Nice has a private nursing branch in Paris. You had better write to the lady superintendent and inquire. It would be folly for any English nurse to go to Paris in the hope of getting work on her own account. There are plenty of women there already engaged in nursing, and, of course, English and American doctors prefer those who are personally known to them. You should not attempt to join any agency or to start "on your own account" without making careful preliminary inquiries. The pay of private nurses is about the same as in London.

(166) *Military* (*Trained Nurse*).—Get form of application from the Army Nursing Department at the War Office, London, S.W., and the Director-General, Medical Department, Admiralty, London.

(167) *Abroad* (*Manchester*).—The General Civil Hospital at Hong Kong was described in The Hospital January 26th and February 23rd this year. The nurses wear uniform, and they are fully trained English ladies. The new matron at Kimberley Hospital has selected suitable washing materials to be worn by her nursing staff. All particulars would be furnished to nurses engaged by either hospital. We cannot advise you to go abroad unless you obtain a definite engagement, or unless you have sufficient private means to make you independent of fees. Even if you took out introductions you might have a long time to wait for private cases. We have often referred to these subjects in our columns. You should look back through your "Nursing Mirror."

(168) *Patent* (*P. E.*).—Inquire of the Secretary, Patent Office, 19, Southampton Buildings, London, W.C. You could go to any invalid furniture manufacturers and see whether your idea has been already carried out. You will find addresses in our advertisement columns.

(169) *Paralysis* (*Jackson*).—You will find addresses of these in "Burdett's Hospital Annual," published by the Scientific Press, 428, Strand.

(170) *Registration* (*Taunton*).—See reply to query (160) last week.

### Wants and Workers.

*Nurse S. E. A.* would be very grateful for help from readers of The Hospital in obtaining for an elderly gentlewoman the pension of £20 granted by the United Kingdom Beneficent Association. So far only 1,611 votes are polled towards the 3,000 required, and others will be thankfully received.

Can any nurse recommend a home in the country for a lady who has occasional epileptic fits? She could pay from 20s. to 25s. per week. Cheerful companionship essential, and plenty of outdoor exercise.—*Miss C.*

### Novelties for Nurses.

### HEALTHY AND COMFORTABLE AIR CLOTHING.

Our own experience, added to that of others, had convinced us that Cellular Clothing was the clothing *par excellence* for summer wear, that its adaptability to all kinds of garments was not sufficiently known, and that something might usefully be done by drawing attention to its good qualities. We, therefore, paid a visit to the warehouses of the company occupied in its sole manufacture. We had heard that many novelties and improvements were being produced continuously, and a personal inspection satisfied us that this was the case. The manager kindly showed us, firstly, all the varieties of the texture itself, and we must describe what cellular cloth really is before proceeding further. Its virtues are obtained through its particular arrangement of texture, it being woven in such a manner as to consist of a number of small cells, running at the back of which are fine threads, which prevent radiation, but which permit of moisture and other substances to escape. Thus, through a scientific discovery, a material has been secured which retains heat and discards all that is unwholesome. Cellular cloth is a pretty fabric, principally made in cotton, but produced in mixtures of silk and cotton and wool and cotton also. It has been held that flannel underclothing was healthier than any other. This is a mistake, especially in respect to closely woven flannels. In the course of wear and washing the flannel shrinks to a dense impenetrable substance, which allows neither a free passage of air or anything else, and so contravenes all principles of health. Even knitted woollen garments shrink in a manner which renders them expensive items in a wardrobe, and the wearing of woollen clothing of any description is nigh to impossible to some sensitive skins. Cellular cloth does not shrink, it is easily washed, remarkably durable, and imparts a sensation of cleanliness to the wearer and does not irritate the skin. All thicknesses are procurable, the very fine, almost like lacework, being prepared specially for wear in India, and the thickest being suitable for the most severe weather in any climate. So much for the material itself. We will now turn to the uses to which it is applied These are, all undergarments for men, women, and children, blouses and tennis dresses, corsets and hats, sheets, bandages, and belts. The underclothing made by the company is tasteful, well worked, and most beautifully finished, so that the minimum of mending is required. All tastes and purses are catered for. The nightgown shown in our illustration is a well-made garment trimmed with strong lace. None but the best trimmings are used, and some of the more elaborate garments for ladies' wear would grace the wardrobe of the most fastidious. A most useful garment is the over-vest of slip, which occupies but a small space, being well shaped to the figure. The cellular corset is a most useful and healthy speciality. It is especially adapted for hot weather, permitting as it does a proper support to the figure to be combined with coolness. There are many forms of ventilated corsets in the market, but we have seen none so good as those supplied by the Cellular Clothing Company. They are prepared with real whalebone, and are shaped after really good models. They retain their shape very well. All varieties are to be had, long and short, and the riding belt is especially suitable to bicycling, a form of exercise which cannot be overlooked nowadays. Those who do not know the cellular cloth we advise to secure a specimen article before summer is over, and to old friends we would point out that the company have produced many novelties, and last, but not least, have lowered their prices. Garments and the materials can be obtained through linen drapers.

Night Gown.

### The Book World for Women and Nurses.

[We invite Correspondence, Criticism, Enquiries, and Notes on Books likely to interest Women and Nurses. Address, Editor, The Hospital (Nurses' Book World), 428, Strand, W.C.]

### MAGAZINES OF THE MONTH.

### "The Pall Mall Magazine."

In the present issue we notice a new departure in certain illustrations which are in colour, and very charming illustrations they are, too. The letterpress of "When Leaves are Green," by "A Son of the Marshes," initiates us into a momentary glimpse of bird life; and we look forward with pleasure to further outcomes from the same pencil and pen. All through the pages of the magazine this month the illustrations are remarkably good. Turn, for instance, to the frontispiece, of the baby suspended cradled in mid air—"Out of Mischief" it is called, and is quite out of the ordinary run of illustration work. "Joan Haste" is continued, as is also "Gwee," by the Marquis of Lorne, and there are some shorter stories, too, complete in themselves. "Evolution in Italian Art" is more educative and solid reading, and here again both letterpress and illustrations are excellent.

### "The English Illustrated Magazine."

If there was nothing else in the June number, one short story in the opening pages of the *English Illustrated Magazine* would sufficiently recommend any journal. This is written by Mr. Barry Pain, and called "A Complete Recovery." "A Sunday Afternoon," by Mr. Henry Shorthouse, is another instance of good fiction—a speciality in which this magazine more especially excels. And without wishing to detract from the merits of fiction, yet somehow one feels in the present instance that a little more solid reading would be an improvement and no drawback to its pages. We must mention that a very good idea is originated in the June issue of a tastily coloured calendar for the month.

### "The Cornhill Magazine."

The chapter on "A Colony for Lunatics" is the most interesting article in the pages of this month's magazine. This is a description, prettily written, of the quaint little Flemish town of Gheel, which in very early days seems to have been a kind of Lourdes. We are told that a certain Saint Dympna, who is buried there—an Irishwoman, by the way—was supposed to have "les faibles d'esprit" under her protection. It was the custom, therefore, throughout the Netherlands, for persons who had insane relations to take them to her tomb, and there offer special prayers for their recovery. The description of the latter day life of lunatics in the town is interesting and well told.

## Figures and Tables

**Figure f1:**